# Childhood Wealth Inequality in the United States: Implications for Social Stratification and Well-Being

**DOI:** 10.7758/rsf.2021.7.3.01

**Published:** 2021-08

**Authors:** CHRISTINA GIBSON-DAVIS, HEATHER D. HILL

**Affiliations:** Sanford School of Public Policy at Duke University, United States.; Daniel J. Evans School of Public Policy and Governance at the University of Washington, United States.

Wealth inequality—the unequal distribution of assets and debts across a population—has reached historic levels in the United States, particularly for households with children (Pfeffer and Schoeni 2016; Saez and Zucman 2016; Gibson-Davis and Percheski 2018). Among households with a resident child under eighteen (child households), levels of wealth inequality are higher than those of income inequality and higher than wealth inequality in other household types (Gibson-Davis and Percheski 2018). In 2019, among child households, those in the top 1 percent of the wealth distribution accounted for 43.5 percent, those in the bottom 50 percent for −0.36 percent.^1^ Given low rates of intergenerational wealth mobility, children who grow up in low-wealth households are likely to have low wealth in adulthood (Pfeffer and Killewald 2018).

Wealth among U.S. child households is notable not only because of its unequal distribution but also because so many child households, particularly those headed by black and Hispanic adults, have so little wealth. Median wealth levels for child households are roughly half as high as those for the general population, and wealth levels for child households in the bottom half of the wealth distribution were −$233 in 2013 (Gibson-Davis and Percheski 2018). Lower-wealth child households are disproportionately nonwhite, with many black and Hispanic child households having next to no wealth. In 2019, median wealth levels were $63,838, $3,175, and $808 for white, Hispanic, and black child households, respectively. These levels reflect long-standing patterns of American structural inequality and institutional racism, under which black and Hispanic families have faced persistent discrimination in lending, credit, and housing markets (Oliver and Shapiro 1995; Darity and Mullen 2020).

As a society, we should be concerned about these trends because wealth may promote child flourishing (Yellen 2016). Disparities in wealth increase gaps in college attendance and completion, and levels of wealth affect early adult decisions regarding marriage and fertility (Conley 2001; Schneider 2011; Addo 2014; Pfeffer 2018). In addition, the importance of wealth is likely not confined to children at the cusp of adulthood but may also operate throughout childhood in multiple domains to affect future life chances (Shanks et al. 2010; Diemer, March- and, and Mistry 2020). For example, wealth has been positively associated with younger children’s standardized test scores (Yeung and Conley 2008; Elliott, Destin, and Friedline 2011) and social-emotional functioning (Shanks 2007; Ream and Gottfried 2019).

Relative to the voluminous literature on income and child well-being, the literature on wealth and child outcomes is still in its infancy, with important contextual and policy issues unaddressed. The association between wealth and some outcomes, such as child health and parenting behaviors, has not been studied, and the moderating effects of race, ethnicity, age, or income have received scant attention. Literature on policies and practices that address economic inequities among child households has largely focused on income-based programs and has provided relatively little evidence as to how policies might address wealth inequities or low-wealth levels among child households.

To further our understanding of wealth and wealth inequality for child well-being, this volume features original theoretical and empirical work on the contours and consequences of wealth for children and their families. The first set of studies describe wealth inequality in the United States, comparing levels of wealth inequality for children across Western democracies and examining parental wealth investments on behalf of their children. The next set examines how wealth affects child health, behavior, and academic achievement, focusing on how wealth interacts with debt and asset composition and income instability. The final set describes current policies and practices that either directly or indirectly boost wealth acquisition among child households.

In this introduction, we lay the foundation for those articles by providing a descriptive and theoretical context for wealth among American children. We describe patterns and trends in wealth and wealth disparities among child households and highlight the extremely low-wealth holdings of African American and Hispanic households. We provide a conceptual framework for how wealth and wealth inequality might matter to children and describe how three aspects of wealth—as a manifestation of resources, conveyance of psychological and economic security, and bestower of class and socioeconomic status—can explain its importance. Last, we briefly summarize the contributions of the articles in this issue before turning to some of their implications for research and policy.

Our conclusions are that growing wealth inequality and the large number of child households with little wealth are concerning aspects of the economic context of childhood in the United States. A relatively small group of parents—mostly white—control the lion’s share of the wealth available to children (Gibson-Davis and Percheski 2018). As with wealth inequality in the population overall, the racial-ethnic disparities in wealth among child households are both profound and profoundly important to address. Our understanding of wealth and its importance to child development is growing but is still hampered by data and methodological challenges. To the extent that we have large-scale wealth policies in the United States, they mostly promote the accumulation of wealth among a small group of families. Interventions to build wealth among families with children or policies to redress wealth inequalities have been scattered and small in scale.

As this issue appears, the United States is still grappling with the economic fallout of the COVID-19 pandemic. Given that the pandemic’s economic effects are disproportionately affecting black and Hispanic households (Hardy and Logan 2020; Federal Reserve 2020b), wealth disparities among child households will continue to grow in the 2020s and many child households will not have enough wealth for their children to flourish. We hope this volume as a whole will contribute to a deeper public understanding of the role of wealth ownership and inequality in child well-being and stimulate advocates and decision makers to create an evidence-based framework for wealth policy.

## DEFINITION OF WEALTH

Wealth, also known as net worth, is a measure of assets minus debts. Assets can be classified as either liquid or nonliquid. Liquid assets can readily be converted into cash via a smoothly functioning market. Common liquid assets include savings or money market accounts, stocks or bonds, and mutual funds. Nonliquid assets require effort to sell and may be sold for less than their value if sold soon after they are acquired. The most common type of nonliquid asset is a home, but other examples include vehicles, ownerships in business, real estate other than a primary residence, and the pre-retirement value of retirement accounts such as 401(k)s or individual retirement accounts. Virtually all Americans (99 percent) report owning some kind of asset (Bhutta et al. 2019) with the most common being a transaction account, that is, checking or savings account (98 percent); owning a vehicle (85 percent); or homeownership (65 percent). Relatively few report owning stocks (15 percent), having business equity (13 percent), or having a savings bond (9 percent) (Bhutta et al. 2019).

Debt also has two primary classifications: secured (backed by collateral that can be collected if the debt is not paid) or unsecured (loaned without collateral). Typical secured debts include car loans and home mortgages; typical unsecured debt include credit card loans and medical debt. Debt has also conventionally been classified as to whether it is “good” or “bad” (Manning and Butera 2010). Good debt will increase future income or wealth. For example, taking on educational loans has conventionally been considered good debt, insofar as human capital acquisition leads to increased earnings, although the recent rise of nonprofit colleges has undermined this traditional classification (Baum 2016). Bad debt is used to acquire something that may depreciate, such as taking out a car loan or using credit cards for consumption purposes. More than three-quarters of Americans report being in debt (Bhutta et al. 2019) and, apart from medical debt, the most frequent types of debt are credit card (44 percent), mortgage (42 percent), and vehicle (37 percent).

Americans’ wealth portfolios depend heavily on the value of their homes. The largest share of the average American asset portfolio comes from the value of a principal residence, which typically constitutes about 25 percent of gross assets (Wolff 2017). Similarly, mortgage debt on the principal residence is the largest category of debt, amounting to some 9 percent of the total (Wolff 2017). When limited to middle-class households, however, homeownership represents an even larger share of the asset and debt pie. For those in the middle three quintiles of wealth, the value of the home and the amount owed on a home are 66 percent and 60 percent of total assets and debts, respectively (Wolff 2017).

Wealth is related to, but conceptually distinct from, income. Wealth is a stock of resources; income is a flow of resources. As a store of value, wealth is what a household can access to meet unexpected expenses, buffer against income loss, or otherwise meet economic shocks, such as medical emergencies, that can deplete financial reserves. As a flow of resources, income can be used to fund day-to-day expenditures and is closely related to a household’s consumption patterns. Wealth is usually built up over long periods, whereas income is used to finance the immediate flow of goods into the household. Unlike income, wealth can be inherited; the intergenerational wealth correlation varies by data sets and methods but is generally thought to be between 0.3 and 0.4 (Pfeffer and Killewald 2018). Because of these conceptual differences, it is perhaps not surprising that wealth and income in the United States are modestly correlated at 50 to 60 percent (Keister and Moller 2000; Killewald, Pfeffer, and Schachner 2017). Among child households, the correlation is 0.50 (Gibson-Davis and Percheski 2018).

## WEALTH IN CHILD HOUSEHOLDS

We begin by describing wealth among child households, using data from the Survey of Consumer Finances (SCF), between 1989 and 2019. The SCF is conducted triennially by the Federal Reserve Board and includes an oversample of wealthy households, making it one of the premier sources of wealth data in the United States (Federal Reserve 2020a). Our analysis updates and expands directly on work by Christina Gibson-Davis and Christine Percheski (Gibson-Davis and Percheski 2018; Percheski and Gibson-Davis 2020). In keeping with that work, we defined child households as those containing at least one member under the age of eighteen (for the technical details on these analyses, see the appendix).

### Wealth Levels

In 2019, child households had median wealth levels of $64,050, roughly half the size of the $114,850 for nonchild households (see table 1). Differences at the median are mirrored in the rest of the distribution; at every point on the distribution, child households had less wealth than nonchild households. Child households at the bottom of the distribution are particularly disadvantaged, both in absolute and relative terms: child household wealth at the 10th percentile was −$20,400 (signifying that these households owed more in debt then they had in assets), 250 percent lower than that of nonchild households (−$8,000).

Asset ownership does not differ substantially by the presence of a child, but debt ownership and amounts do (see table 2). Similar percentages of both child and nonchild households report having any asset, owning a home, having a retirement account, or owning stocks, bonds, or mutual funds. Median asset levels are also similar (about $200,000) but child households have higher median home values, whereas nonchild households have more in retirement and stock accounts. In contrast, child households are more likely to report being in debt than nonchild households (87 percent versus 72 percent), and, conditional on being in debt, have higher levels of median debt ($122,000 versus $47,500). Part of the reason child households are more indebted is their home debt: more than half of child households report owing money on their homes (versus one-third of nonchild households), with median loan amounts running some $160,000 (about 50 percent more than the median amount for nonchild households). Differences in credit card debt ownership and levels are relatively small, but child households are twice as likely to report owning money on education loans (31 percent versus 17 percent). Conditional on having educational loans, loan amounts are higher for nonchild households ($27,000 versus $18,000).

Lower levels of wealth for child households, relative to those without resident children, is consistent with the life-cycle model, the conventional lens used to understand life course dynamics in wealth (Ando and Modigliani 1963). The model, which connects individuals’ debt and asset patterns to expectations of future income, predicts that younger-aged households (typically those with resident children) will have lower levels of wealth than older (typically those without resident children). Young adults, who may have high education debt and low current earnings, will not save but will instead borrow against future income. Adults in middle to late-middle age, at the peak of their earnings capacity, will pay down debts and accrue assets and savings at an increased pace. Older adults (usually interpreted to be those over the age of sixty-five, or past the typical age of retirement) will spend down their savings to support consumption in the absence of labor-market activity (Modigliani 1988). The life-cycle model can also partly explain low levels of intergenerational wealth mobility. Wealth that is not used in one generation is passed to the next, providing that generation with a “leg up” on asset accumulation, relative to households that did not receive an inheritance (Gale and Scholz 1994).

### Wealth Inequality

**Figure 1.**
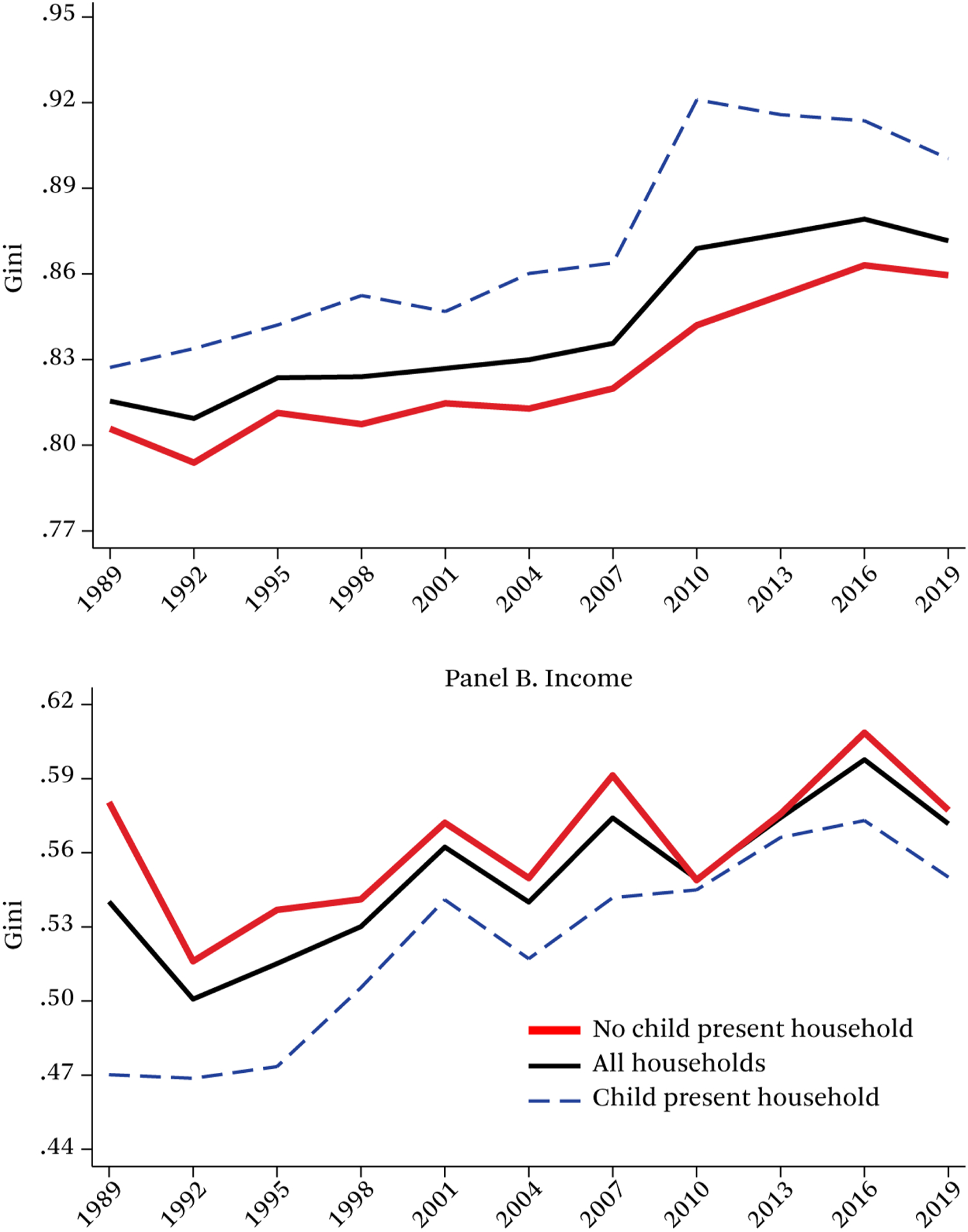
Gini Coefficient, Net Worth and Income, 1989–2019 *Source:* Authors’ tabulation based on the Survey of Consumer Finances (Federal Reserve 2020a).

As measured by the Gini coefficient, child households in the United States have more and faster growing wealth inequality than households without a child present (see figure 1). From 1989 through 2019, wealth inequality rose for all households, particularly during the Great Recession (2007–2009) and immediately after. However, across all years, child households, relative to households without a resident child, had higher levels of inequality, with larger year-over-year increases in the Gini, especially during the Great Recession. As a result, the relative gap in the Gini coefficient between households with and without a child increased between 1989 and 2019. In 2019, the Gini coefficient for child households was 0.90 and for households without a resident child, 0.86. Notably, this pattern does not hold for income inequality: across the period, the income Gini for child households was lower than that of nonchild households. Income inequality has also fallen for child households over time.

As shown in table 3, the wealth distribution for child households is extremely top heavy, with net worth increasingly concentrated among the very wealthiest. In 2019, the top 10 percent of the wealthiest child households accounted for 82 percent of all wealth among child households, the top 1 percent accounting for 43.5 percent. Relative to 1989, wealth in the top decile increased by 14 percentage points, with most of that increase (11 percentage points) occurring in the top percentile. The increasing concentration among the top decile was at the expense of those in the bottom 90 percent, where the concentration of wealth fell steadily over time. Declines in wealth were particularly pronounced for households in the bottom 50 percent of the distribution—wealth shares never rose above 1 percent and by 2019 were negative. Notably, the distribution of wealth among nonchild households is less extreme than among child households. Across years, relative to the top 10 percent of child households, the top decile of nonchild households accounted for a smaller fraction of overall wealth. Although wealth share in the bottom 50 percent of nonchild households declined (from 2.2 percent in 1989 to 0.96 in 2019), wealth shares for the group were nevertheless positive (albeit very small).

The extreme and growing concentration of wealth seen among child households does not extend to income. The top 10 percent of richest households account for a smaller share of income (47 percent in 2019) than the top 10 percent of wealthiest households do for wealth (82 percent). And, unlike share of wealth, shares of income by place in the distribution remained relatively stable over time. Finally, in contrast to child households in the bottom 50 percent of the wealth distribution, which had no wealth, those in the bottom 50 percent of the income distribution accounted for approximately 14 percent of the income pie. Relatively larger increases in wealth inequality than in income inequality for child households is consistent with trends for the general population (Gibson-Davis and Percheski 2018; Wolff 2018) and may reflect the snowballing effect of income inequality (Saez and Zucman 2016). Those with the highest incomes save at disproportionately high rates, leading to higher asset accumulation. These assets beget more income (in the form of capital income, such as a stock dividend), further exacerbating disparities in wealth and income shares (Saez and Zucman 2016; Saez 2017).

Why is wealth inequality more extreme in child households than other households? It is likely that the factors that have led to increases in wealth inequality for U.S. households more generally—a shifting labor market, changes in tax codes that favor wealth accumulation, and growing levels of household indebtedness (Saez and Zucman 2016, 2019)—have been particularly acute for child households. Child households typically rely on earnings as their primary source of income, but globalization and a skill-based workforce have caused wages to stagnate for lower- and middle-class workers as they sky-rocketed for upper-class workers (Autor, Katz, and Kearney 2008; Autor 2014). When coupled with a tax code that has become increasing regressive, top earners can save more of their incomes, have bigger stores of reserves to deal with unexpected expenses, and have more after-tax monies to invest in assets (Saez 2017; Saez and Zucman 2019). Wealth inequality has also risen because Americans are taking on increasingly large levels of debt (Saez 2017). Mortgage debt has increased over time, though it has slowed in recent years (Haughwout et al. 2019) and pressure to buy more expensive homes may be more acute for child households than those without children, as school choice is closely tied to neighborhood location. The proportion of adults of childbearing age who have taken on education loans has also risen (Houle 2014; Addo, Houle, and Sassler 2019) because adults increasingly take on education loans to finance higher education for themselves.

Two other factors may explain greater wealth inequality among child households than other households. First, the effects of the Great Recession were particularly large on younger household heads with fewer years of labor-market experience and households of color (Hoynes, Miller, and Schaller 2012; Pfeffer, Danziger, and Schoeni 2013), characteristics that may describe a substantial fraction of child households. Second, child households have become increasingly diverse in terms of race-ethnicity and family structure (England, Wu, and Shafer 2013; Child Trends 2016), both of which are strong predictors of wealth (Oliver and Shapiro 1995; Yamokoski and Keister 2006; Percheski and Gibson-Davis 2020).

### Demographic Disparities in Wealth

**Figure 2.**
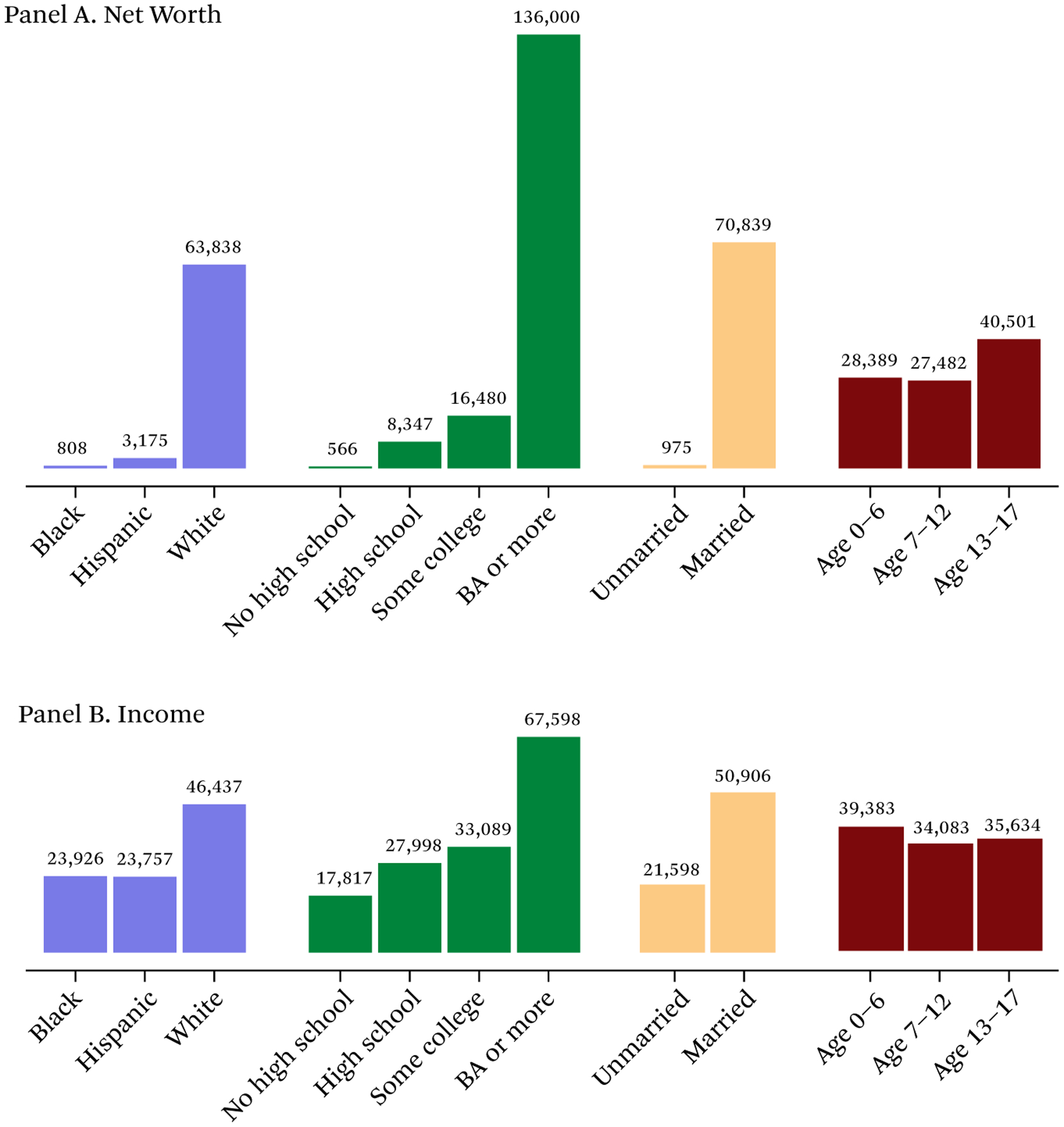
Median Net Worth and Income, Select Characteristics, Child Households, 2019 *Source:* Authors’ tabulation based on the Survey of Consumer Finances (Federal Reserve 2020a). *Note:* Age refers to age of oldest child.

Consistent with previous work (Percheski and Gibson-Davis 2020), wealth levels among child households are characterized by alarmingly large racial and ethnic disparities (figure 2). In 2019, white child households had median wealth levels of $63,838, compared with black child household levels of $808. Expressed as a ratio, these estimates indicate that, at the median, for every dollar of white child household wealth, black child households had 1 cent. Relative to African Americans, Hispanic child households had substantially higher levels ($3,175), but still only 5 cents for every dollar of white wealth.

Educational gaps in wealth were also large; those with a bachelor’s degree had median wealth levels 240 times as high as those without a high school degree ($136,000 versus $566). Differences by marital status were less pronounced than education, but still quite large: children living with married parents had median wealth levels ($70,839) seventy-two times as high as those living with unmarried parents ($975). Differences by age of the oldest child are relatively smaller and, most likely reflecting that older households’ heads have older children, indicate that wealthiest child households are those with teenagers. Wealth disparities by socioeconomic characteristics exceed those seen for income (figure 2, panel B).

**Figure 3.**
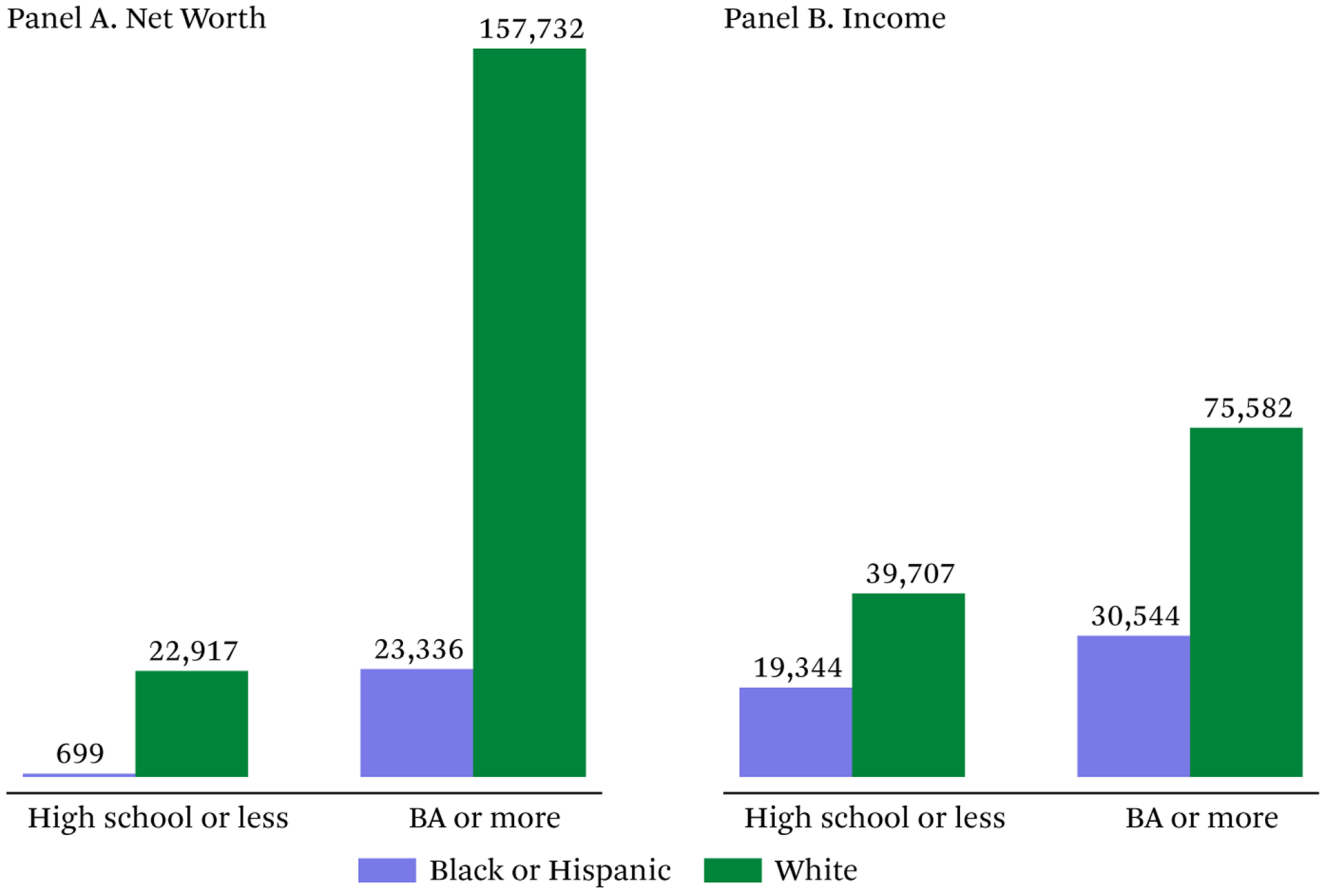
Median Net Worth and Income, Race-Ethnicity and Education, Child Households, 2019 *Source:* Authors’ tabulation based on the Survey of Consumer Finances (Federal Reserve 2020a).

Notably, racial-ethnic differences in wealth persist even after adjusting for socioeconomic status, as proxied by educational attainment (figure 3). Among households where the head has a high school degree or less, white child households have median wealth levels thirty-two times higher than those of black or Hispanic child households. Among those with bachelor’s degree or more, white child households have wealth levels that are six and a half times as high. The racial-ethnic discrepancy in wealth levels among the most educated is so vast that black and Hispanic child households with at least a bachelor’s have wealth levels ($23,336) that only slightly exceed that of whites that did not graduate high school ($22,917).

With regard to specific asset types (table 4), white child households are far more likely to hold each asset and to have much higher median values if they do, compared to black and Hispanic child households. White child households have median asset levels that are fourteen times as high as that of black households and six times as high as Hispanic families. Notably, the share of child households receiving an inheritance are substantially higher for white (21 percent) child households than black (3 percent) or Hispanic (7 percent) child households.

White child households have three to four times higher levels of debt than black or Hispanic households as well as high levels of mortgage debt. However, white child households are the only subgroup with a positive asset-to-debt ratio (about 2, or $2 in assets for every dollar of debt). Black child households have 59 cents for every dollar of assets, and Hispanic child households almost break even ($0.93 asset to $1 debt). Relative to white and Hispanic child households, black child households report relatively low levels of mortgage and credit card debt, but high levels of education debt.

The vast wealth advantage that white parents enjoy reflects not only modern day structural and cyclical economic changes but also U.S. history and the effects of past public policies (Oliver and Shapiro 1995; Conley 1999; Darity and Mullen 2020). The amassing of wealth among white families began with the institution of slavery and the appropriation of Native lands. It continued during Reconstruction with the failure of the Freedmen’s Bureau to reallocate land to free slaves and then with federally supported covenants and “redlining” against nonwhite and non-Christian families during the suburbanization of America in the post–World War II era (Oliver and Shapiro 1995; Conley 1999; Rothstein 2017).

These historical forces are the antecedents of contemporary barriers to wealth accumulation among Americans of color (Darity and Mullen 2020). Despite antidiscrimination laws, households of color continue to face discrimination in obtaining credit, purchasing homes, and securing loans (Oliver and Shapiro 1995). Residential segregation results in black and Hispanic families occupying neighborhoods that have lower housing values and higher rates of vacancy and foreclosure (Massey 2015). Redlining has been replaced by “reverse redlining,” in which lenders target racial minority households for subprime loans and other predatory loan practices (Rugh and Massey 2010; Faber 2013). Policies associated with mass incarceration and the use of fines and fees in the criminal justice system disproportionately affect black families and impede wealth accumulation by lowering family incomes, imposing legal costs, and incurring debts (Harris, Evans, and Beckett 2010; Sykes and Maroto 2016; Nepomnyaschy et al. 2021, this issue). Black and Hispanic children are also disadvantaged in that they are less likely to receive inheritances or in-vivo transfers from their parents (Killewald and Bryan 2018; Pfeffer and Killewald 2019). Relative to whites, black and Hispanic parents provide fewer monetary resources to their adult children (Fingerman et al. 2011; Hardie and Seltzer 2016), likely because parents of color have fewer financial resources to offer (Oliver and Shapiro 1995). Although inheritances explain only a small fraction of the intergenerational transmission of wealth (Pfeffer and Killewald 2018), racial and ethnic differences in parent-to-child transfers of wealth contribute to and compound the difficulties black and Hispanics face in accumulating wealth (Pfeffer and Killewald 2019).

## HOW WEALTH MATTERS TO CHILDREN

Research on wealth and its consequences for child well-being has largely concentrated on outcomes in young adulthood, and in particular, wealth’s association with educational attainment (Shanks 2007; Elliott, Destin, and Friedline 2011; Jez 2014; Doren and Grodsky 2016; Ream and Gottfried 2019; Diemer, March- and, and Mistry 2020). After adjusting for household income, household wealth is positively associated with years of completed schooling, high school graduation, college attendance, and college completion (Conley 2001; Belley and Lochner 2007; Pfeffer 2011; Jez 2014; Doren and Grodsky 2016). Many of these studies rely on wealth data collected in the 1970s and 1980s, before wealth inequality began to rise overall and among child households. However, Fabian Pfeffer’s (2018) recent study on wealth and educational attainment by birth cohort suggests that wealth may be an increasingly important predictor of human capital acquisition. Comparing children born in the 1970s with those born in the 1980s, he finds that the difference in college attainment between the lowest and highest quintiles of wealth had increased by nearly 10 percentage points. The wealthiest children born in the 1980s were 40 percentage points more likely to graduate from college than their least wealthy peers.

A smaller literature has examined wealth as a predictor of outcomes for children under the age of eighteen. Studies published before this issue indicate that parental wealth is positively correlated with standardized test scores and academic achievement (Orr 2003; Yeung and Conley 2008; Friedline, Masa, and Chowa 2015), an association that cannot be explained by income alone (Yeung and Conley 2008; Diemer, Marchand, and Mistry 2020). A handful of studies examine children’s social-emotional functioning, finding that more wealth is associated with higher sociability in eight-grade students (Ream and Gottfried 2019) and fewer behavior problems in three-through twelve-year-olds (Shanks 2007).

Enhancing this narrow research base is new work presented in this volume demonstrating the importance of wealth in buffering the adverse effects of low income on child achievement (Miller et al. 2021), the negative associations between wealth and child body mass index (BMI) (Boen, Keister, and Graetz 2021), and the adverse repercussions of fathers’ child support arrears for children’s socio-behavioral outcomes (Nepomnyaschy et al. 2021). It also offers evidence that, despite wealth’s strong association with cognitive outcomes, black-white and Hispanic-white disparities in test scores remain even in same-wealth families, in part because of demographic and wealth composition differences (Conwell and Ye 2021). These studies represent important contributions to our understanding of the importance of wealth for children. Nevertheless, relative to the voluminous literature on income and child development (Duncan and Brooks-Gunn 1997; Duncan, Magnuson, and Votruba-Drzal 2014; Shonkoff 2018), the literature on wealth and child development is in its nascent stage.

### Conceptual Framework

**Figure 4.**
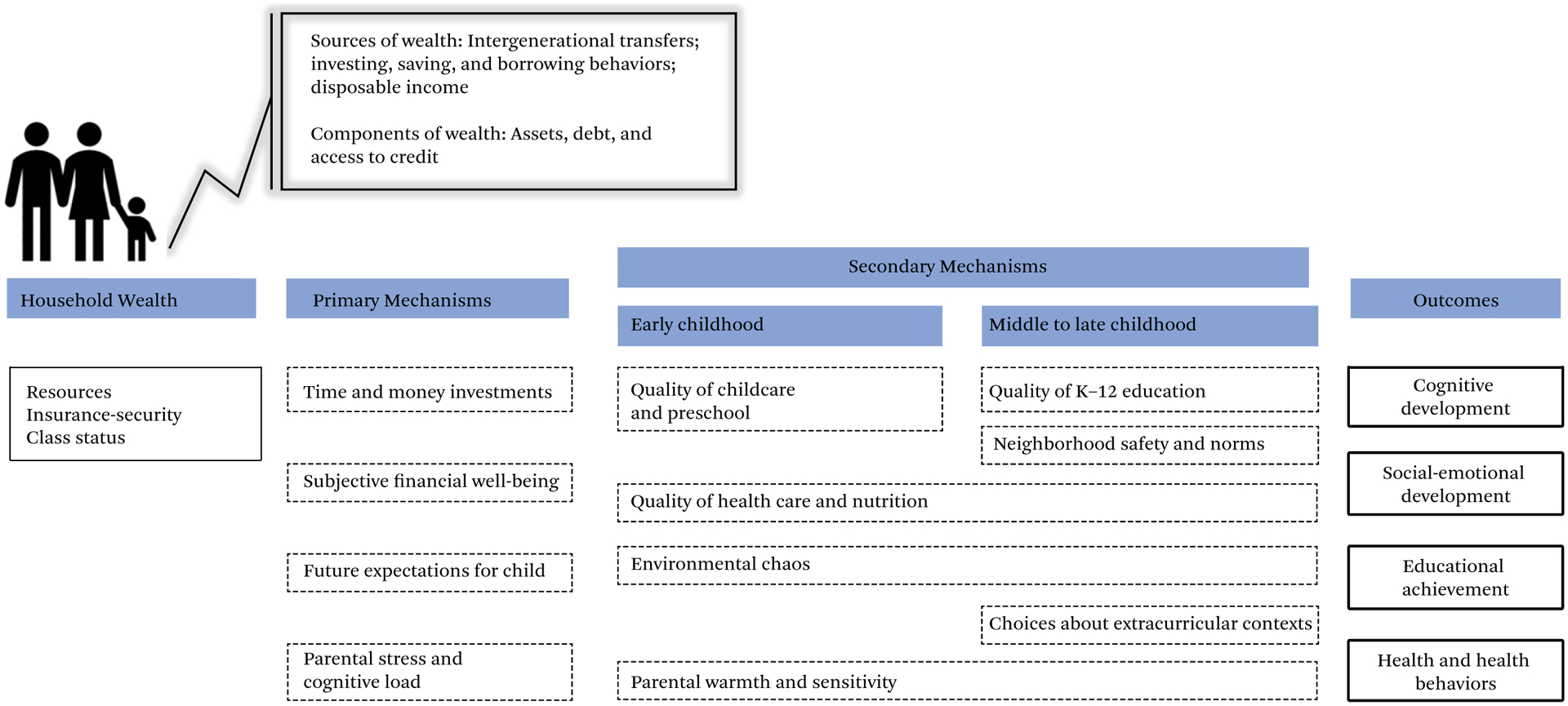
Hypothesized Mechanisms for the Effects of Household Wealth on Child and Adolescent Outcomes *Source:* Authors’ compilation.

Despite the relative paucity of empirical studies on wealth inequality and child development, it is theoretically likely that wealth is a determinant of many aspects of child well-being. In figure 4, we describe the potential mechanisms linking wealth with child outcomes. We begin by listing three key meanings of wealth for families: resources, insurance-security, and class status.

First, although wealth represents a stock of resources, it can contribute to both income and expenses. Some assets produce interest or dividends, which are returned to the owner as income, and parents can use this to provide additional resources to their child. For instance, interest on a savings account might be treated by parents the same as any other income source. Conversely, debt may constrain parental investments, if paying back loans depletes financial resources or compromises parenting ability by increasing levels of anxiety and stress.

Second, wealth can provide a family with the real and psychological security of having insurance against crises (Shapiro 2004; Pfeffer 2018). This security comes in large part from wealth being less attached to labor-force participation than income (Shanks and Destin 2009) and therefore providing a buffer against unexpected changes in job status, earnings, or health crises (Sherraden 1991; Shapiro 2004). The insurance function of wealth may also allow parents to take financial risks (such as investing in education) that will pay off in the long run.

Third, far more than income, wealth expresses class status. As Dalton Conley (1999) notes, wealth both represents and determines class: assets and debt are the visible evidence of class status and the mechanisms used to change it. In fact, the value of one’s home and the real and perceived quality of the neighborhood in which one resides is probably the strongest demonstration of class and mobility. The typical connotation of the American dream—of “making it”—is a house on a leafy street with a picket fence. For immigrants, homeownership is also evidence of incorporation in the economy and culture of the arrival country (Myers and Lee 1998; Sánchez 2018; Agius Vallejo and Keister 2019). This symbolism gives wealth particular meaning for the subjective experience of social and economic position, opportunity, and belonging. In addition, symbols of class status shape how individuals—including children—are treated by others and perceive themselves in ways that influence behavior, academic achievement, and health (Goodman et al. 2000; Destin et al. 2012; Destin 2013; Mistry et al. 2015).

How do the resources, security, and status provided by wealth affect family life and other contexts of child development? We hypothesize a set of primary and secondary mechanisms that could mediate relationships between family wealth and child outcomes (see figure 4). Our model is informed by the theories linking family income to child development (McLoyd 1990; Conger and Elder 1994; Mayer 1997; Bradley and Corwyn 2002; Yeung, Linver, and Brooks-Gunn 2002). Two of the mechanisms—parental investments and parent stress—are those most commonly associated with income effects on children. However, the other two—subjective financial well-being and future expectations—are likely to be particularly salient for understanding the role of wealth in child development.

#### Time and Money Investments

Liquid wealth can be used like income to invest in food, housing, health care, and schooling, which can then benefit children in a variety of ways. The need to pay down debt can constrain the use of income for basic needs, but mortgages and educational debt may represent investments in better quality neighborhoods or careers. The influence of wealth via parental investments would most likely be seen in the quality of child care and education, the neighborhood context in which families live, and the degree of instability or “chaos” experienced at home and in the community (Shanks 2007; Evans and Wachs 2010). The evidence that liquid wealth is more predictive than illiquid wealth of school-age children’s test scores (Yeung and Conley 2008) supports the notion that parents are using wealth for consumption related to child development. As indicated by scholarship on poverty (see Yeung, Linver, and Brooks-Gunn 2002), the effects of wealth that operate through parental investments may be stronger for health and cognitive development than for social and emotional outcomes. Wealth may also affect the choices that parents make about work outside the home, which could influence both the quantity and quality of parent-child time.

#### Parental Stress and Cognitive Load

Akin to the strong theoretical connections between income and parental stress (McLoyd 1990, 1998; Conger and Elder 1994), wealth and parental stress are likely also linked. Levels of wealth could inform a parents’ comprehensive assessment of the resources available to provide basic needs, achieve goals, and support family processes. In fact, though untested, the ability of wealth to offer insurance against income losses suggests that it may be far more effective at reducing parental stress about finances than income is. Even nonliquid wealth, such as homeownership or the ownership of a retirement fund, is likely to shape the sense of day-to-day sufficiency of resources. Substantial evidence shows that parental stress leads to less warm and sensitive parenting, which in turn is associated with children’s behavior problems (Conger et al. 1994; McLoyd 1998; Mistry et al. 2002).

Also, recent developments in behavioral science focus on the way in which income poverty increases cognitive load and demands attention, improving some cognitive functions and demeaning others. For example, some studies find associations between having insufficient resources and poor performance on intelligence tests, better memory for costs, and a greater ability for considering financial trade-offs (Mani et al. 2013; Shah, Shafir, and Mullainathan 2015; Shah et al. 2018). Having no or negative wealth could further add to cognitive load; savings or assets could reduce it. As insurance against income losses, assets could reduce the cognitive demands of being poor or low income.

#### Subjective Financial Well-Being

We hypothesize that wealth could also affect parent and child subjective financial well-being (SFWB). Consistent with scholarship, we define SFWB as a broad set of emotions and attitudes about current and future finances, including future orientation and optimism, risk aversion, and sense of financial control (Vera-Toscano, Ateca-Amestoy, and Serrano-Del-Rosal 2006; Zyphur et al. 2015). SFWB is an inherently comparative concept, in which individuals compare their circumstances both with others and with their experiences and expectations (Vera-Toscano, Ateca-Amestoy, and Serrano-Del-Rosal 2006). Although untested, SFWB among child households could more likely be affected by wealth than income or other measures of socioeconomic status. Higher net worth may increase parents’ future orientation and optimism while decreasing their risk aversion, all of which could lead to reduced stress and greater investments in children. Higher debt, however, could increase future orientation in a way that increases stress and decreases optimism.

#### Future Expectations for Children

More than income, wealth may influence parent and child expectations about children’s education and future trajectories. In a process he calls “the construction of future possibilities,” Michael Sherraden (1991, 152) hypothesizes that assets promote future orientation by creating real and perceived opportunity, which is internalized by both children and their parents. Empirical studies provide support for Sherraden’s hypothesis: homeownership and other assets are often more predictive of parental expectations for their children’s education than income is (Zhan and Sherraden 2003). Experiments in psychology also suggest that wealth may positively shape children’s aspirations for college or other goals (Destin and Oyserman 2009). The positive relationship between wealth and college outcomes is likely mediated through parental expectations (Shanks and Destin 2009; Elliott, Destin, and Friedline 2011), and parental expectations may be a stronger explanatory factor for the effects of wealth on educational attainment than parental investments (Diemer, Marchand, and Mistry 2020). Consistent with this mechanism, Jin Huang and colleagues (2021, this issue) provide evidence that child development accounts raise parental expectations about their child’s educational future.

#### Potential Moderators

Informed by life course and developmental theory, our model hypothesizes that some mechanisms are likely more relevant during certain developmental stages than others. For example, although wealth could reduce parental stress and improve parental warmth and sensitivity at any point, those effects might be most important in early childhood when children are developing secure attachments and foundational cognitive and behavioral skills. Likewise, parental optimism, and parent and child expectations about the future, may be of particular significance as children begin the transition to young adulthood and make choices about their post-secondary-education paths. The few studies on wealth effects by child age have provided conflicting evidence as to whether effects vary by age (Yeung and Conley 2008; Diemer, March- and, and Mistry 2020). In one of the first studies to use a developmental lens to examine changes in wealth and outcomes at different points in childhood, Portia Miller and colleagues (2021, this issue) hypothesize that the effects would grow with age but find consistent beneficial effects of wealth on cognition and behavior regardless of age.

Other potential moderators include family size, wealth composition, and wealth levels. Given the same wealth, a larger family will have fewer resources per child than a smaller family. Yet some of the hypothesized benefits of wealth, including insurance and class status, are not divisible. For instance, class status is likely determined by total wealth and attributed equally to all children regardless of number. Among other things, these attributes of wealth make it unclear whether it should be adjusted for family size in studies describing wealth during childhood (for a larger discussion on whether estimates of wealth should be adjusted by household size, see Killewald, Pfeffer, and Schachner 2017). Also, different components of wealth (debt, homeownership) may have different qualitative meaning to families, suggesting that a family’s wealth composition may moderate the effects of wealth on children. For instance, unsecured debt has been associated with negative child outcomes in part because it increases stress (Berger and Houle 2019). However, debt may also signify increased opportunity for children, as would happen if parents take on increased mortgage debt to secure access to higher quality schools or safer neighborhoods (Conwell and Ye 2021, this issue). Finally, the marginal value to parents and children of an additional $1 in wealth likely diminishes because wealthy families may derive less utility from increases in net worth than less affluent ones. The association between wealth and child well-being may also be non-linear if families have achieved a specified threshold of wealth (for example, to weather a short-term job loss or to fund a college degree). To date, however, we are not aware of any studies that have investigated the marginal utility or threshold effects of wealth.

## CONTRIBUTIONS TO THIS VOLUME

The volume begins with two articles describing the contours of wealth for American child households. First, to understand child household wealth inequality in the global context, Fabian Pfeffer and Nora Waitkus conducted the first cross-national comparison of wealth among children using the Luxembourg Wealth Study. Consistent with the life-cycle hypothesis of wealth accumulation (Modigliani 1988), they find that across the fourteen countries studied, children have higher levels of wealth inequality than the elderly. Additionally, the degree of wealth inequality experienced by a country did not correlate with their level of income inequality. Notably, the United States stands out in this analysis for having exceptionally high levels of wealth (and income) inequality among child households.

The next study describes a uniquely twenty-first-century dimension of wealth inequality—“financially intensive parenting”—a term Nina Bandelj and Angelina Grigoryeva coin to describe how some American parents are increasingly directing more resources toward child-centric savings and borrowing. Their analyses using the SCF show that parental investing, saving, and borrowing for children differ by family wealth and parent race-ethnicity. White parents with wealth above median levels accumulate relatively high levels of child-centric assets, whereas black and Hispanic parents across the wealth distribution accumulate (by comparison) fewer child-centric assets, and for black parents, higher levels of educational debt. They conclude that increasing disparities in financially intensive parenting likely contribute to inequality in educational opportunities for children by economic circumstances and race-ethnicity.

Moving from contours to consequences, the next set of studies considers how wealth and its unequal distribution may contribute to child well-being. Evidence of the protective effects of wealth is found in Courtney Boen, Lisa Keister, and Nick Graetz’s examination of the associations between wealth and child BMI using the Panel Study of Income Dynamics (PSID). They find that higher levels of parental wealth, even after adjusting for a host of conventional socioeconomic status markers (such as maternal education, income, health insurance status), were associated with lower BMI and decreased obesity risk. The results were consistent across types of assets and debts, except for home equity, which was negatively associated with child BMI. The effects of wealth on child BMI operate not only directly, as they do in adults, but also indirectly through household spending and family stress processes. This study offers important new evidence on the linkages between wealth and population health disparities.

Using the National Longitudinal Survey of Youth 1979 (NLSY79), Jordan Conwell and Leafia Zi Ye investigate racial and ethnic gaps in achievement for households with the same levels of wealth. They find that, in families with the same net worth, black and Hispanic children have lower standardized math and reading scores relative to white children. Racial disparities in achievement for same-wealth families were explained both by racial-ethnic differences in the demographics of same-wealth families and in the composition of wealth. Notably, relative to white families with the same net worth, black and Hispanic families had fewer liquid assets and less “good” debt, such as housing debt. These results highlight the nuanced ways in which economic and racial-ethnic disadvantage may intersect and the potential benefits of certain types of debt for purchasing “developmentally advantageous school and neighborhood contexts.”

Further underscoring the importance of debt type is the study by Lenna Nepomnyaschy and colleagues, which examines unsecured debt, including credit card debt, loans, and child support arrears, among children with nonresident fathers. Using the Fragile Families and Child Wellbeing Study, the authors find that nonresident fathers’ child support arrears, which are often not measured in other surveys about wealth, were a large proportion of nonresident fathers’ debt. In addition, child support arrears were associated with decreased socioemotional functioning for nine- and fifteen-year-olds. Notably, these negative associations did not hold for other types of unsecured debt, suggesting that child support arrears play a unique (but heretofore overlooked) role in the lives of low-income children.

Consistent with the hypothesis in the conceptual framework that wealth serves as a source of security, Portia Miller and coauthors provide the first evidence that wealth may be protective for low-income children. In their investigation of the associations between parental wealth and child outcomes at three developmental stages (preschool, middle age, and adolescence), wealth was positively correlated with cognitive achievement and negatively correlated with behavioral problems at all three time points. The estimated effects of wealth were also larger than the effects of income. Notably, among households with low levels of income, wealth was associated with better outcomes for children, perhaps because these families could draw on the resources that wealth offers. An implication of their work is that children in families that are both low wealth and low income are at particular risk of poor outcomes.

The final set of studies examine policies that, either directly or indirectly, promote asset accumulation. Jin Huang and colleagues describe one of the few U.S. policy models for increasing savings in low-income child households, Child Development Accounts (CDAs), illustrated by the Saving for Education, Entrepreneurship, and Downpayment program in Oklahoma (SEED OK). After summarizing the impacts of SEED OK, which increased family educational savings and reduced maternal depression and punitive parenting practices, the authors propose ten design features of universal, progressive, and lifelong CDAs. Seven states have already adopted this policy model in some form, and more are likely to follow, making the insights of these authors valuable to guiding future policy development.

Examining the possibility that income-support programs have positive spillovers on wealth accumulation, Katherine Michelmore and Leonard Lopoo estimate the effects on wealth of the Earned Income Tax Credit (EITC) using the PSID. They find that exposure to the EITC during early childhood (up to five years) increases familial wealth during middle childhood (six to ten years), particularly among families with a less-educated head of household. These effects operated through increased funds in checking, savings, and retirement accounts, and higher amounts of home equity. Greater EITC exposure was also associated with higher amounts of credit card debt. The authors find some suggestive evidence that the program increases low-income families’ position in the wealth distribution as well as their individual wealth level.

Margo Jackson, Chinyere Agbai, and Emily Rauscher use simulated Medicaid eligibility to investigate how state-level generosity in Medicaid affects wealth among families with children. Using the NLSY79, the authors find that an increase in prenatal and infant eligibility of one standard deviation—roughly equivalent to the difference between Massachusetts and Oklahoma in the early 1990s—was associated with a 22 percent increase in savings and retirements amounts. Medicaid eligibility was also associated with higher average home mortgage amounts, but not home value, overall net worth, or other forms of debt. In addition, the positive associations with savings and mortgages were only evident for non-Hispanic white and more highly educated mothers. The unequal results by demographic group suggest that increasing eligibility for Medicaid may exacerbate, rather than mitigate, existing differences in wealth among child households.

### Research Implications

The work presented in this volume address some of the most pressing questions regarding wealth and child well-being, but many unknowns remain. We still know relatively little about how wealth varies across a child’s life course, and we are only beginning to understand whether the importance of wealth in determining child well-being varies by developmental stage (Miller et al. 2021; Nepomnyaschy et al. 2021). Emerging evidence also suggests that assets and debts may operate differently in the lives of children (Conwell and Ye 2021), but we do not yet understand the theoretical or mechanistic reasons why this is so. Additionally, research has yet to address how household wealth intersects with salient contexts of the child’s life because most studies operationalize wealth as acting independently of factors such as school quality or neighborhood context. Although it is clear that wealth inequality for American children far outstrips that in other countries (Pfeffer and Waitkus 2021), the social and policy levers that account for that differential are unknown. Finally, despite some emerging evidence, the work on how policies affect levels of wealth for children is sparse.

These questions could be more readily answered if scholars did not face data limitations in measuring the contours and consequences of wealth for children. Analysts must choose between data sources that fully capture the American wealth distribution but offer no data on child well-being (the SCF), and data sources that collect information on child outcomes but omit the very wealthiest households (the PSID and NLSY). The lack of the very wealthiest households is a notable omission given that American wealth for child households is even more highly skewed than wealth among the general population (Gibson-Davis and Percheski 2018). In the absence of complete wealth data, scholars have used homeownership or home equity, which is available in a lot more surveys, as a proxy for wealth (Pfeffer 2018). Doing so, however, ignores variations in wealth among nonhomeowners. This incomplete measure is particularly problematic for analyses of racial-ethnic disparities in wealth, given that more than half of Hispanic and two-thirds of black child households do not own homes (Percheski and Gibson-Davis 2020). Extant data sources also omit information on citizenship and nativity status, which are likely to be salient to wealth among child households, particularly Hispanic ones.

In addition, currently available data do not include several types of debt for child households, including child support arrears, criminal justice debt, and medical debt. As Nepomnyaschy and coauthors demonstrate in their article in this volume, child support arrears are the primary source of debt for low-income fathers. Outside the Fragile Families and Child Wellbeing Study these authors use, however, no data source of which we are aware collects data on child support arrears. Only one survey, the Survey of Household Economics and Decision-making, has collected data on legal debt, adding these questions in 2019, but the data cannot be disaggregated to families with children. The development of new data sources or expansions of existing ones with an eye toward valid and reliable measurement of all possible components of wealth and of multiple domains of child well-being would be extremely valuable.

To the extent possible, analysts should also consider specific components of wealth that are most salient for their research questions. Child household net worth portfolios are heterogeneous, with households varying in the types of assets and debts they hold, as well as the relative amounts of assets versus debts (Conwell and Ye 2021, this issue). Households that are asset heavy versus those that are debt heavy may differ in their constraints and psychological resources (Berger and Houle 2016, 2019). Additionally, asset and debt ownership vary by race and ethnicity, with white households being more likely to have the kinds of debts that might promote child well-being (mortgage-related debt). Variation in asset and debt ownership, when coupled with an endogenous association with race and ethnicity, complicates the analytic task as to how wealth relates to child well-being (Conwell and Ye 2021, this issue).

A final consideration for analysts is whether they are concerned about inadequate levels of wealth or inequities in wealth ownership. The wealth distribution among child households is characterized by both low levels of wealth for many and an astoundingly large gap between those at the top and those at the bottom. Studies on children over eighteen suggest that both wealth scarcity and wealth inequality are important (Pfeffer 2018), but work focusing on this topic for children under eighteen is scant.

The articles in this volume highlight several possible avenues for addressing key methodological challenges in operationalizing and analyzing wealth inequalities. For instance, the lack of overlap in the wealth distribution between white and black or Hispanic households can create common support problems that bias estimates or make them imprecise. Articles in this volume deal with this challenge by using fewer comparison groups (see Bandelj and Grigoryeva 2021) or comparing racial and ethnic differences for households with the same levels of wealth, such as at the 25th percentile (Conwell and Ye 2021). Analysts focusing on wealth and child well-being also face methodological challenges that plague observational studies on economic scarcity more generally. Establishing causality with observational data is difficult, and most analyses of wealth are limited to descriptive relationships. Even experimental and quasi-experimental studies that leverage policy shocks to income or wealth often test interventions that could change both income and wealth, making it difficult to disentangle the effects of each separately. Huang and colleagues (2021) offer a promising example of interventions designed to change wealth without changing income.

### Policy Implications

The United States has never had a cohesive policy approach to wealth. Relative to that on income poverty and inequality, for example, far less policy consensus exists as to whether wealth inequality or insufficient wealth ownership are problems worthy of government intervention. Not surprisingly, then, no wealth analog exists for the multitude of income-based safety net programs at the federal, state, and local levels that offer cash assistance, in-kind goods, and direct services. The hodgepodge of policies that do affect asset and debt accumulation include financial regulations, tax policies, and small, local, asset accumulation programs. President-elect Joe Biden has indicated support for several policies that would directly address wealth, including plans to provide relief from college debt, the establishment of “Baby Bonds,” a child development account given to all children in the United States at birth (Hamilton and Darity 2010), and higher taxes on corporations and capital gains. Biden’s plans would be the most comprehensive political effort to address wealth inequality, but it remains to be seen how many will come to fruition.

A key step to analyzing the policies related to wealth and wealth inequality is to agree on a definition of the problem: Are we most concerned about wealth disparities or inadequate wealth ownership? Does the kind of wealth matter? Should we promote asset accumulation or debt reduction? And should we worry about equalizing wealth by race, ethnicity, or social class, or attacking the underlying structural and racial problems that lead to such disparities? Depending on our problem definition, policies could be designed to promote multiple goals, including building savings and assets in low-wealth families, or in families of color; reducing “bad debt”; and reducing extreme wealth by individuals and firms. Some evidence is provided in this volume on the value of specific interventions for asset accumulation among low-wealth families, including both asset-building and income-support programs (Jackson, Agbai, and Rauscher 2021; Michelmore and Lopoo 2021; Huang et al. 2021). Notably, interventions to reduce inequality would need to be far more ambitious, targeting both the top and the bottom of the wealth distribution and redistributing wealth systematically and intergenerationally.

Defining the problem, setting the goals of wealth policy, and deciding on specific policy tools require not only more empirical evidence, but also agreement on the values and political interests of the American people. We offer three considerations to stimulate that discussion. First, our current policy approach to wealth promotes and protects wealth accumulation at the top, but this orientation is relatively recent. In the middle of the twentieth century, the heyday of the American Dream—at least for white, Christian Americans—income and estate tax rates were both around 70 percent and corporate tax rates were around 50 percent. Currently, many forms of income generated from wealth are tax free (Tax Policy Center 2020); corporate taxes are set at historically low levels (21 percent) and relatively easy to avoid (Saez and Zucman 2019); and tax-exempt investment vehicles in retirement and education primarily benefit higher-income families (Pressman and Scott 2017; Toder, Khitatrakun, and Boddupalli 2020). Individual estates are exempt from taxes up to a jaw-dropping $11.6 million. In addition to those to tax wealth, proposals exist to limit or replace tax exemptions and deductions with credits that benefit low- to middle-income families (Gale 2017; Toder, Khitatrakun, and Boddupalli 2020).

Second, policies to address wealth inequality must consider the trade-offs between universal versus targeted approaches, a key tension in U.S. social policy generally. Universal approaches garner more political support, less stigma, and fewer concerns about disincentivizing work; targeted approaches are better at reaching those most in need and addressing inequities (Ellwood 1988). Current U.S. policies designed to address poverty and income inequality mix these approaches, as exemplified by Social Security (universal) and the EITC (targeted). These tensions will exist in the context of wealth interventions as well. In this issue, Huang and colleagues (2021) suggest that CDA accounts should take a universal approach. Another possibility is *targeted universalism*, a relatively recent framework in which goals are universal but interventions are targeted to the specific needs and burdens of historically marginalized groups (powell, Menendian, and Ake 2019). In terms of reducing wealth inequality, targeted universalism may point the way to more explicit acknowledgement of the role of racism and racist policies in the creation of black-white and Hispanic-white wealth gaps, and to more targeted interventions aimed at offering black and Hispanic families more opportunities to accumulate and keep wealth.

Third, asset limits in cash assistance programs are a barrier to wealth accumulation and social mobility. Most U.S. public assistance restricts eligibility to families without significant wealth. These policies are designed to cull families from the assistance rolls who have low income but substantial wealth. In practice, relatively few families fall into this category and asset tests (as these requirements are called) act as a direct barrier to low-income families’ economic mobility (Ratcliffe et al. 2016). Owning a car or house or having college savings for a child can make a family ineligible for food assistance, the EITC, Medicaid, and other programs, which disincentivizes wealth accumulation. This problem could be solved administratively by removing asset limits or setting them at much higher levels. In the 1990s and 2000s, many states loosened asset tests for the Temporary Assistance for Needy Families and Supplemental Nutrition Assistance Program (SNAP). The evidence on the effects of these reforms on asset accumulation are mixed, but it has been noted that raising asset limits for one program (such as SNAP) may not be sufficient because most low-income families receive multiple means-tested benefits all with different asset limits (Nam, Ratcliffe, and McKernan 2008).

## CONCLUSION

We are gaining a deeper and more troubling understanding of the contours of wealth for American children. As a country, America stands apart from other Western democracies, insofar as levels of child household wealth are distributed far more unequally than in other parts of the globe (Pfeffer and Waitkus 2021, this issue). Racial and ethnic disparities are profound, with black and Hispanic families having pennies on the dollar for every dollar of white wealth. Mechanisms to promote wealth building—homeownership, savings, and stock participation—are characterized by structural and racial inequities that keep them out of the hands of many (Bandelj and Grigoryeva 2021, this issue; Conwell and Ye 2021, this issue). Policies and practices to build wealth among families with children have been scattered and diffuse, and some policies may inadvertently increase wealth inequality rather than reduce it (Jackson, Agbai, and Rauscher 2021, this issue; Michelmore and Lopoo 2021, this issue).

Following prior scholarship, we argue that the relevance of wealth to families and children is threefold: wealth provides resources, security, and class status. These theoretical linkages between wealth and child well-being overlap with, but remain distinct from, those of income. The conceptual difference is most obvious in terms of security: income, which can fluctuate considerably within years and months, likely does not engender the same feelings of economic and psychological security that wealth provides. But the conceptual differences between the two are evident in other ways. The physical manifestations of wealth—most notably, homeownership, and neighborhood choice—shape child opportunities and expectations that exist independently from income (Chetty and Hendren 2018). Perhaps more than they do income, parents may use their levels of wealth as cues to their child’s future life chances (Huang et al. 2021, this issue) and shape their current parental investment choices accordingly.

Given the role that wealth plays in family functioning, it is no surprise, then, that wealth is related to child outcomes (Orr 2003; Yeung and Conley 2008; Friedline, Masa, and Chowa 2015). As demonstrated in this volume and elsewhere, lower levels of parental wealth are correlated with lower levels of academic performance, worse physical health, impaired social and behavioral outcomes, and fewer years of completed schooling (Pfeffer 2018; Diemer, Marchand, and Mistry 2020; Boen, Keister, and Graetz 2021, this issue; Miller et al. 2021, this issue; Nepomnyaschy et al. 2021, this issue). Wealth effects remain after accounting for income, and the association between wealth and child well-being may be larger in magnitude than the association between income and child well-being (Miller et al. 2021, this issue). Wealth appears to be beneficial for children across developmental stages and across racial-ethnic identities (Diemer, Marchand, and Mistry 2020; Conwell and Ye 2021, this issue; Miller et al. 2021, this issue), but equalizing wealth would not be enough to eliminate race- and ethnic-based gaps in achievement (Conwell and Ye 2021, this issue).

Wealth inequality among child households is rising faster than among the general population, showing no indication that the pace will slow. A relatively small group of parents—mostly white—control the lion’s share of wealth that is available to children (Gibson-Davis and Percheski 2018). A sizable share of American children, and a large majority of black and Hispanic children, grow up in households with very low levels of wealth and are likely to have low levels of wealth as adults. Wealth appears to be critical to the successful flourishing of children (Miller et al. 2021, this issue), yet policies and practices are just beginning to grapple with the consequences of wealth’s inequitable distribution. Equalizing wealth among white and black children may not be enough to eliminate racial disparities in academic achievement, but the current gaps are without question adversely shaping the life experiences and chances of black and brown children. We call on scholars, policymakers, and practitioners to address the unequal distribution of wealth among children in the United States and to understand that unabated increases in wealth inequality are likely to have negative repercussions for all of American society, but particularly for black and brown children. We hope this volume sparks even greater research study and engagement with advocates and decision makers that will inform a coherent framework for wealth policy.

## Figures and Tables

**Table 1.** Net Worth, 2019, by Presence of Child in the Household

Percentile	Net Worth		
	All Households	No Child	Child^a^
10th	−11,849	−8,000	−20,400
25th	1,600	3,700	300
50th	97,300	114,850	64,050
75th	372,420	425,000	265,400
90th	1,157,000	1,238,300	892,000

*Source:* Authors’ tabulation based on the Survey of Consumer Finances (Federal Reserve 2020a).

*Note:* All estimates weighted.

a Household has at least one resident child under eighteen.

**Table 2.** Select Asset and Debt Holdings, by Presence of Child in the Household, 2019

	Any		Home		Retirement		Stocks or Bonds	
Assets	Has	Median	Has	Median	Has	Median	Has	Median
**All households**	.99	201,200	.65	225,000	.50	65,000	.25	22,000
Without resident child	.99	200,620	.65	210,000	.48	73,000	.26	28,000
With resident child	.99	204,250	.65	250,000	.55	53,000	.24	10,000
	Any		Mortgage		Credit Card		Educational	
Debts	Has	Median	Has	Median	Has	Median	Has	Median
**All households**	.77	65,000	.42	135,000	.45	2,700	.21	22,000
Without resident child	.72	47,500	.36	109,000	.43	2,500	.17	27,000
With resident child	.87	122,000	.55	160,000	.52	3,000	.31	18,000

*Source:* Authors’ tabulation based on the Survey of Consumer Finances (Federal Reserve 2020a).

*Note:* Medians expressed in dollars. Median conditional on having the asset or debt. Results weighted.

**Table 3.** Share of Wealth, by Presence of a Child in the Household, Wealth Percentiles, and Year

	All Households				No Child Present				Child			
	0–50	51–90	91–99	100	0–50	51–90	91–99	100	0–50	51–90	91–99	100
**Wealth**												
1989	1.68	29.0	38.1	31.2	2.22	28.8	38.9	30.0	0.64	30.8	36.1	32.4
1995	1.90	27.0	34.0	37.1	2.56	27.4	33.5	36.5	0.90	26.2	35.9	37.0
2001	1.72	26.7	38.2	33.4	2.38	27.2	37.7	32.7	0.76	25.8	39.2	34.2
2007	1.50	25.3	38.4	34.8	2.32	25.8	39.1	32.8	0.18	24.3	36.7	38.9
2013	−0.07	22.8	40.4	36.8	0.76	24.5	40.1	34.6	−1.41	18.9	40.2	42.3
2019	0.55	21.1	39.9	38.5	0.96	22.2	40.3	36.6	−0.36	18.4	38.5	43.5
**Income**												
1989	15.7	42.0	25.3	17.0	19.4	44.8	23.8	12.1	13.8	39.4	26.4	20.3
1995	16.8	43.9	25.0	14.3	19.1	45.4	24.9	10.6	15.9	42.3	24.9	16.9
2001	15.0	39.6	25.4	20.0	16.1	40.4	24.9	18.6	14.7	38.7	25.7	21.0
2007	14.6	38.3	25.8	21.3	16.3	39.2	25.3	19.2	13.8	37.4	26.2	22.7
2013	14.5	38.6	27.2	19.7	15.0	38.2	27.2	19.5	14.4	38.7	27.2	19.7
2019	14.6	38.9	27.4	19.1	15.7	39.5	26.9	17.9	14.3	38.6	27.4	19.7

*Source:* Authors’ tabulation based on the Survey of Consumer Finances (Federal Reserve 2020a).

*Note:* Estimates represent share of wealth attributed to percentile group. Estimates weighted.

**Table 4.** Select Assets and Debts, Child Households, by Race and Ethnicity

Assets	Any		Home		Retirement		Stocks or Bonds		Inheritance	
	Has	Median	Has	Median	Has	Median	Has	Median	Received	Median
White	1.00	291,901	.77	260,000	.66	65,000	.32	10,000	.21	55,000
Black	.99	20,970	.40	177,000	.37	15,700	.12	9,000	.03	23,000
Hispanic	.97	45,900	.48	200,000	.30	21,000	.05	3,300	.07	35,000
Debts	Any		Mortgage		Credit Card		Educational			
	Has	Median	Has	Median	Has	Median	Has	Median		
White	.93	147,100	.67	164,000	.53	3,500	.34	17,200		
Black	.76	35,000	.31	136,000	.48	1,900	.39	22,000		
Hispanic	.78	49,000	.39	145,000	.55	2,000	.17	14,000		

*Source:* Authors’ tabulation based on the Survey of Consumer Finances (Federal Reserve 2020a).

*Note:* Medians expressed in dollars. Median conditional on having the asset or debt. Results weighted.

## Footnotes

1. Authors’ calculations from the Survey of Consumer Finances. See table 3 for data on wealth percentiles and figure 2 for data on median math levels by race-ethnicity.

## APPENDIX

Data are from the Survey of Consumer Finances (SCF), a repeated cross-sectional study of U.S. households conducted by the Federal Reserve triennially. The SCF is considered the best source of wealth data in the United States because it includes both a nationally representative sample of households and a sample of high-income households designed to represent the distribution of net worth (Keister 2014; Kennickell 2008). Because the SCF’s unit of analysis is the primary economic unit, which is akin to the census definition of a household, we refer to our unit of analysis as households. We used ten SCF waves between 1989 and 2019 and report monetary estimates in 2019 dollars.

The primary sample consisted of households in which the household contained at least one member under the age of eighteen. Of those under eighteen, 98 percent were designated as a child of the respondent or their spouse’s child; we refer to these household members as children. This definition excludes households with children who reside elsewhere and may include households with resident children over eighteen.

We define net worth as the value of total household assets less total debts. Assets include the value of savings and checking accounts, certificates of deposit, pooled investment accounts, stocks, bonds, retirement accounts, the value of the primary residence and other real estate, business assets, tangible assets (such as art and jewelry), and assets not classified elsewhere. Debts include mortgages on the primary residence, mortgages on other real estate, business debt, credit card debt, educational debt, vehicle loans, and other liabilities.

Following previous work (Wolff 2018), our measure of net worth excludes the future value of pension and Social Security income, assets that a household may realize in the future but does not currently possess. We also exclude the value of a household’s vehicles because such vehicles typically have a high consumptive value (for example, are necessary transportation for work) and cannot realistically be converted to cash.

Our descriptive analysis stratified our analysis by the following covariates. Race-ethnicity measured whether the head of the household was non-Hispanic white, non-Hispanic black, or Hispanic (the SCF’s public use version does not include any additional racial or ethnic categories). Marital status measured whether the head was married (1 = married). Education of the head consisted of four categories, measured dichotomously: no high school diploma, high school diploma (omitted category), some college, bachelor’s degree or more. Age of the oldest child in the household was measured in the following ranges: younger than six, seven to twelve, or twelve to seventeen (continuous measures of child’s age were not available). Family income included earnings, rents, alimony or child support, and all other types of income.

Our first analyses present a number of descriptive measures on wealth and wealth inequality, presented for the full sample, and then stratified by the presence of a resident child. We then present descriptive statistics on the ownership and presence of assets and debts, and, conditional on the presence of that asset or debt, the median amount held or owed. We also examine the amount of wealth held by families at different parts of the distribution. The last set of analyses focuses on child households and examines how wealth levels vary by the sociodemographic characteristic of the household head, as well as asset and debt ownership by the race and ethnicity of the household head. All analyses were adjusted for survey design and survey nonresponse using SCF sampling weights.
